# Individualizing therapy – in search of approaches to maximize the benefit of drug treatment (II)

**DOI:** 10.1186/1468-6708-5-7

**Published:** 2004-08-16

**Authors:** Cornel Pater

**Affiliations:** 1 Hanover, Germany

## Abstract

Adjusting drug therapy to the individual, a common approach in clinical practice, has evolved from 1) dose adjustments based on clinical effects to 2) dose adjustments made in response to drug levels and, more recently, to 3) dose adjustments based on deoxyribonucleic acid (DNA) sequencing of drug-metabolizing enzyme genes, suggesting a slow drug metabolism phenotype. This development dates back to the middle of the 20^th ^century, when several different drugs were administered on the basis of individual plasma concentration measurements. Genetic control of drug metabolism was well established by the 1960s, and pharmakokinetic-based individualized therapy was in use by 1973.

## Patterns of drug prescription

Despite tremendous advances in the science and technology of drug development, as well the emergence of guidance and consensus building among scientists, many clinicians, pharmacists, and consumers remain uninformed regarding the scientific basis of establishing bioequivalence, the generic-drug approval process, and the issues related to individualizing therapy in general [7,8]. The consequence may be drug dosing errors: overdosing or underdosing of drugs, resulting in the occurrence of harmful effects or the nonoccurrence of the expected treatment benefit.

Recent information [9] indicates that doctors are not consistently prescribing proven treatments at recommended doses, and at times they are not prescribing proven treatments at all. A decrease in dose may decrease the efficacy (*relative risk reduction *[RRR]) of therapy and thereby decrease the treatment's *net benefit*. Not prescribing an agent will effectively nullify the potential benefit to individuals, and when repeated frequently enough, failure to prescribe the agent will significantly decrease the benefit to the population as a whole. At other times, doctors tend to prescribe a drug more generally than clinical trials dictate. The treatment of a population with lower *outcome prevalence *(OP) decreases net benefit and may lead to *harm*. Overdosing may increase treatment-related harm, and underdosing may erode efficacy; both will result in diminished treatment benefits. Finally, noncompliance on the part of the patient may lead to a decrease in efficacy and a requisite decrease in net treatment benefit. If a patient reduces the dose without totally eliminating the drug, the risk of non-dose-related side effects of treatment may remain.

The relationship between the terms mentioned above, which govern treatment success, can be expressed mathematically as follows [10-12]:

Net Benefit = RRR * OP - Harm

**Figure 1:**
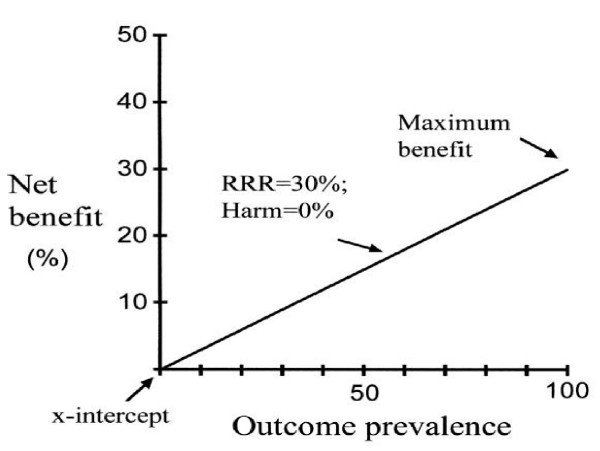
Basic relationships. Net benefit is plotted as a function of outcome prevalence. The *line *represents the relationship with the assumption of a relative risk reduction of 30% and treatment harm of 0%. The *line *is the maximum net benefit that can be attained at any given outcome prevalence. The *x-intercept*, or benefit threshold, represents the outcome prevalence at which net benefit will accrue to individuals and the population as a whole. The point of maximum benefit occurs when the outcome prevalence is 100%; at this point, if harm is absent, the net benefit or efficiency of treatment equals the relative risk reduction or efficacy of that treatment. Benefit decreases proportionately as a function of outcome prevalence.

The graphical representation in Figure 1 allows for a series of observations that expand our understanding of the benefits and risks of treatment.

The difficulties in drug prescription that are mentioned above are not related to the physicians' training or experience but result instead from difficulties in relying with confidence on label claims of efficacy, safety, and interchangeable use of new drugs "within class." The common denominator among these problems seems to be an insufficiently well crystallized knowledge base regarding the proper use and interpretation of general terms such as *equivalence/similarity, pharmaceutical equivalence/therapeutic equivalence*, and *bioequivalence/bioavailability*. Problems are further compounded by the increasing use of generic drugs and the interpretation of such terms as *prescribability and switchability*.

## Term definitions

*Bioavailability *(BA) indicates a measurement of the rate and amount of therapeutically active drug that reaches the general circulation and its presumed site of action [13].

### Bioequivalent drug products (BE)

Bioequivalence is the absence of a significant difference in the rate at which, and the extent to which, the active ingredients in pharmaceutical equivalents become available at the site of drug action in the body when administered under similar experimental conditions in an appropriately designed study. A product may also be considered bioequivalent to an innovator product if (a) the difference in rate of drug absorption between the two products is intentional and (b) no significant difference is found in the extent of absorption of the two products when they are evaluated under similar experimental conditions [13-17].

*Bioequivalence requirement *refers to a requirement, imposed by the Food and Drug Administration (FDA), of in vitro and/or in vivo testing of specified drug products that must be satisfied as a condition of marketing [17].

*Pharmaceutical alternatives *are drug products that contain the identical therapeutic moiety, or its precursor, but not necessarily in the same amount or dosage form as the same salt or ester. Each such drug product individually meets either the identical or its own respective compendial or other applicable standards of identity, strength, quality, and purity, including potency and, where applicable, content uniformity, disintegration times, and/or dissolution rates [17].

### Pharmaceutical equivalence

To be considered pharmaceutically equivalent, two drug products must (a) contain identical amounts of the same active ingredients in the same dosage form, (b) be formulated to meet the same compendial or other applicable standards of quality and purity, and (c) generally be labeled for the same indications. However, pharmaceutical equivalents may differ in the excipients (e.g., flavors, preservatives) that they contain, as well as in their shape, scoring, packaging, and in certain circumstances, their labeling [17].

*Average bioequivalence *involves assessment of pharmacokinetic parameters such as area under the curve (AUC) and peak concentration (Cmax), as well as calculation of a 90% confidence interval for the ratio of the averages of these parameters for the two products that are compared, usually a test product (T) against a reference product (R). The calculated confidence interval should fall within a conventionally established BE limit of 80% to 125% for the ratio of the product averages [18]. The clinical judgment underlying this BE limit is that a test product with BA measures outside this range would be denied market access. However, in specified circumstances, clinical judgment permits widening or narrowing of the BE limit (e.g., 90% to 111% for narrow-therapeutic-range drugs and drug products).

Since the implementation of the FDA Bioavailability and Bioequivalence Requirements in 1977, the assessment of bioequivalence has been a subject of continuous debate [16,20-22]. This controversy has led to modifications in the bioavailability/bioequivalence regulations and guidelines. Despite these modifications, the process of assessing bioequivalence continues to evolve as scientific consensus emerges on many of the issues driving the debate [23].

The average BE reflects comparison of population averages and therefore fails to assess the subject-by-formulation interaction variance (i.e., the variation of the averages in particular individuals). In contrast, the newer *population *and *individual *approaches reflect differences in the objectives of BE testing at various stages of drug development. These differences are embodied in the concepts of *prescribability *and *switchability *(interchangeability) [19-22].

These concepts underscore the difference between the population and individual bioequivalence approaches. Population bioequivalence assesses total variability of the measure in the population, and it becomes important when physicians are initially prescribing a medication and they need to rely on the average performance of the drug product [24]. In contrast, the most important consideration for individual bioequivalence rests on the assurance that products deemed bioequivalent can be used interchangeably in the target population (i.e., they exhibit switchability) [25]. In addition to the comparison of averages, the individual bioequivalence approach compares within-subject variabilities and assesses subject-by-formulation interaction. It offers flexible equivalence criteria based on the individual therapeutic window and variability of the reference drug product. Furthermore, it allows scaling criteria for highly variable/narrow-therapeutic-range drugs and promotes the use of subjects from the general population in bioequivalence studies.

*Prescribability *refers to the clinical setting in which a practitioner prescribes a drug product to a patient for the first time. In this setting, the prescriber relies on the understanding that the average performance of the drug product has been well characterized and relates in some definable way to the safety and efficacy information from clinical trials. *Switchability *refers to the setting in which a practitioner transfers a patient from one drug product to another. This situation arises with generic substitution, as well as with postapproval changes by an innovator or a generic firm in the formulation and/or manufacture of a drug product. Under these circumstances, both the prescriber of the drug and the patient should be assured that the newly administered drug product will yield safety and efficacy comparable to that of the product for which it is being substituted. However, such a switch may, in fact, occur without the patient's or clinician's knowledge, and this concern is addressed in equivalence studies designed to minimize the risk to the patient in both situations.

**Figure 2:**
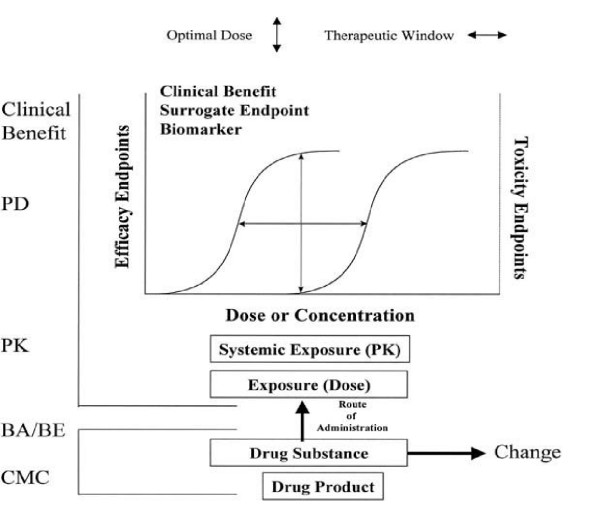
Exposure-response relationships. Relationships between (1) drug substance and drug product, (2) exposure expressed as dose or systemic exposure on log scale, and (3) positive (efficacy) or negative (toxicity) outcomes. These outcomes may be measured by clinical end points, surrogate endpoints, or biomarkers. The relationships between exposure and outcomes define the optimal dose and therapeutic window. The term *change *introduces the concept of equivalence in outcomes before or after a specified change (eg, generic substitution, postapproval manufacturing change). CMC, Chemistry, manufacturing, and controls; BA/BE, bioavailability and bioequivalence; PK, pharmacokinetics; PD, pharmacodynamics.

Although average bioequivalence is the recommended parameter for most bioequivalence studies, the current FDA guide titled Statistical Approaches to Establishing Bioequivalence [26] recommends that population and individual bioequivalence also be evaluated in some cases. Understanding of this process is enhanced by the outline in Figure 2, which illustrates the classical exposure-response relationship that might assist in adjusting dosages and dosing regimens in the presence of influences on pharmacokinetics (PK) by demographic factors (e.g., age, gender), intrinsic factors (e.g., impaired organ function), or extrinsic factors (e.g., concomitant medication, food intake).

The outcomes (clinical benefit, reflecting the "response" component of the relationship), can be measured as clinical or surrogate endpoints or as biomarkers. The relationship between exposure and outcomes, expressed as dose- or concentration-response curves that have efficacy and toxicity levels at their extremes, define the optimal dose and the therapeutic window [27]. Theoretically, these curves should be generated in different individuals for developing prescribability criteria, and in the same individuals for developing switchabiliy criteria.

## Premarketing risk-benefit assessment

The approach described above is a simplified framework of successive steps to be taken in phases I and II of the development of any drug, with the aim of generating data for drug labeling (i.e., data on dose-response [effectiveness and toxicity] relations of the new agent and how these depend on patient characteristics). The dose regimen of such a new agent, explored and established through phases I and II, is to be demonstrated in phase III as "safe and effective" for the claimed indication. Proper study design and research methodology, as well as appropriate statistical analysis, should be applied to ensure that the drug's effectiveness can be substantiated. Further, the outcome derived from two such randomized clinical trials should document that the estimate of the true treatment effect favors the new drug over the reference drug and that the toxicity of the new agent does not exceed acceptable limits. In other words, a first risk/benefit assessment would favor the new drug.

Having come that far, a question justifiably arises: what logic leads to the conclusion that the new drug is likely to be effective in future patients? That is, are the treatment benefits generalizable to an actual clinical population? The question is extended to *external validity*, meaning the extent to which the conclusions of a study would hold true for other persons, in other places and at other times. Examples from most therapeutic areas indicate that approved drugs may not work as expected when applied in broad community populations, in real-world settings, and among diverse practitioners operating under real-world constraints [28]. In such instances, years of effort and huge investments fail to meet the intent of providers, the expectations of consumers, or the demands of healthcare payers.

Conventionally, the dose-response trials mentioned above, exploring the behavior of biomarkers both cross-sectionally between individuals treated with different doses of an agent, and longitudinally within individuals as doses (or concentrations) change with time, supply the necessary information on the drug's pharmacologic action (the so-called *empiric confirmation *at a conventional *α *level). However, despite all their methodological rigorousness, these trials may not entirely eliminate factors that influence bioavailability in earlier development stages and internal validity in later-phase studies (randomized controlled trials [RCTs]).

Bioavailability may be influenced by

• Patient-related factors, such as concurrent diseases, differences in first-pass metabolism, interactions with concomitant medications, diet, circadian biorhythms, the influence of fed-versus fasted-state physiologic conditions, and gastrointestinal factors (e.g., pH, motility, blood flow, bacterial flora) [29-32].

• Product-related factors, such as: physical and chemical properties of the drug (e.g., solubility, degree of ionization, crystalline forms, chemical form, isomers), as well as variables related to manufacturing, formulation, or both (e.g., coatings, compression force, particle size, presence or absence of excipients) [30-33].

To control for as many variables as possible, most bioequivalence trials are conducted with healthy volunteers as subjects, [19,34,35] and in real patients only in circumstances wherein the use of volunteers would be unethical (e.g., tests with cytotoxic drugs) or when assessment of bioequivalence is based on pharmacodynamic and/or clinical end points [35].

The *causal confirmation *is much more complex than the empiric confirmation, even if the drug's previously established pharmacologic action is believed to ensure that the drug has the same intrinsic property that alters the clinical outcome – in the RCT patients – in a similar way and to a similar extent as in the previous patients.

A number of other causes, however, may interfere in the conduct phase of an RCT. These causes include

• Confounding – a distortion of an association between an exposure and disease brought about by extraneous factors.

• Interaction – the interdependent operation of two or more factors that produce an unanticipated effect.

• Transience – an idiosyncratic property of a drug that displays its expected pharmacologic property when tested in one batch, but not in others.

Many of the specific design and analysis features applied to RCTs (e.g., blinding, randomization, intention-to-treat analysis) are meant to ensure that the possibility of confounding is minimized or eliminated. The same does not apply in the case of transience or interaction, for which independent evidence is needed to eliminate those possibilities.

## Highly variable drugs

Drugs that tend to exhibit high degrees of variability in their pharmacokinetic profiles are known to complicate the assessment of bioequivalence [22,36,37]. This is, at least in part, a function of the high intrasubject variability (previously defined as greater than 30%) of the drug or drug product [22]. Examples of such drugs are propafenone immediate release, verapamil, and nadolol.

## Narrow-therapeutic-index drugs

Small changes in systemic concentration of such drugs can lead to marked changes in pharmacodynamic responses [19-22]. A broader term for the narrow-therapeutic-index drugs is "critical-dose drugs." Characteristically, these agents require blood-level monitoring, need to be dosed on the basis of body weight or other individualized parameters, display serious clinical consequences if overdosing or underdosing occurs, and manifest a steep dose-response relationship [38].

A typical drug in this category is warfarin, which is widely used for its anticoagulant properties. For the most part, metabolism of S-warfarin occurs by means of the gene CYP2C9 [39]. Inhibition of this isoform results in several clinically important drug interactions. Fluconazole, metronidazole, miconazole, and amiodarone are a few examples of the many drugs that profoundly inhibit S-warfarin metabolism and produce marked increases in prothrombin time (PT) measurements [40-43]. A multitude of endogenous and exogenous factors may contribute alone or in combination to either increasing or decreasing PT ratio or the INR response [44].

Factors that increase PT ratio or international normalized ratio (INR) response include:

Endogenous factors: 11

Exogenous factors: 117 specific drugs and 49 different classes of drugs

Factors that decrease PT ratio or INR response include:

Endogenous factors: 5

Exogenous factors: 42 specific drugs and 24 different classes of drugs

Physician surveys have indicated that most clinicians favor more rigid bioequivalence guidelines for these types of drugs [8]. Others have recommended that the bioequivalence requirement for these agents be based on intrasubject variability, as well as the pharmacokinetic-pharmacodynamic relationship. Although the FDA has not modified the bioequivalence guidelines for critical-dose drugs, the Canadian regulatory authority, Health Canada, has narrowed the CI requirement for these drugs to 90% to 110%. For prescribability, the current requirements may be adequate for all drugs, including those with a narrow therapeutic index. However, some clinicians have expressed concerns about switchability [7,39]. Several reports suggest that once a patient has been carefully titrated on a narrow-therapeutic-index drug, the formulation should not be switched [7,8]. The same might be true of warfarin.

Drug products suspected of having bioequivalence problems are listed in the Orange Book (Approved Drug Products with Therapeutic Equivalence Evaluations) [45]. Such drugs may exhibit a narrow therapeutic index or solubility problems, or they may be poorly absorbed or unstable in gastrointestinal fluids [33]. Among clinicians there seems to be widespread concern that even small changes in the bioavailability of drugs' active ingredients might lead to significant changes in the efficacy or safety of those products [7,8,38].

## The concept of risk and its application to drug development

In light of serious concerns about risks incurred from using medical products, a variety of public and private agencies involved in health care are dedicating more attention to examining the current system of managing these risks. The main goal is to focus on the costs and value of better data concerning the incidence and causes of injuries from medical products and the roles of all stakeholders in risk management.

The FDA has issued a concept paper [46] presenting preliminary thoughts on risk issues, including:

• Important risk assessment concepts.

• Generation and acquisition of safety data during product development.

• Analysis and presentation of safety data in an application for approval.

*Risk assessment*, defined as the process of identifying, estimating, and evaluating the nature and severity of risks associated with a product, should be continuous throughout the life cycle of any product, whereas the process of *risk management intervention *is intended to enhance the safe use of a product by reducing risk.

With regard to the current trend toward *systemic risk confrontation*, the FDA, Center for Drug Evaluation and Research (CDER), and Center for Biologics Evaluation and Research (CBER) appear ready to develop and finalize, by fall 2004, guidance documents regarding risk assessment, clinical pharmacovigilance, and risk management. Table 1 highlights the components of a risk management system (RMS).

**Table 1:** Components of a Risk Management System

• Risk Assessment – estimation & evaluation of risk
• Risk Confrontation – determination of acceptable levels of risk in societal context
• Risk Intervention – actions to control risk
• Risk communication – interactive process of exchanging risk information
• Risk Management Evaluation – measurement of effectiveness of aforementioned activities

## Conclusions

Determining the optimal initial dosage regimen (prescribability) and maintaining safety and efficacy outcomes when the regimen is changed in some way (switchability) demand careful decision making in the application of equivalence approaches. These approaches must be applied differently during the three phases of the drug development process, and the knowledge-base that is derived from this process must be transferred to and utilized by physicians and pharmacists to assist them in prescribing and dispensing medicines to patients. Consistent and appropriate management of equivalence approaches supports good assessment, management, and communication about risks associated with a therapeutic product, as expressed in product labeling, as well as in specifications and standards that control the quality of a therapeutic product in the marketplace.
